# Peptide Extracts from Native Lactic Acid Bacteria Generate Ghost Cells and Spheroplasts upon Interaction with *Salmonella enterica*, as Promising Food Antimicrobials

**DOI:** 10.1155/2020/6152356

**Published:** 2020-10-05

**Authors:** Gabriela N. Tenea

**Affiliations:** Biofood and Nutraceutics Research and Development Group; Faculty of Engineering in Agricultural and Environmental Sciences, Technical University of the North, Av. 17 de Julio s-21, Barrio El Olivo, 100150 Ibarra, Ecuador

## Abstract

Protecting foods from contamination applying peptides produced by lactic acid bacteria is a promising strategy to increase the food quality and safety. Interacting with the pathogen membranes might produce visible changes in shape or cell wall damage. Previously, we showed that the peptides produced by *Lactobacillus plantarum* UTNGt2, *Lactobacillus plantarum* UTNCys5-4, and *Lactococcus lactis* subsp. *lactis* UTNGt28 exhibit a broad spectrum of antibacterial activity against several foodborne pathogens *in vitro*. In this study, their possible mode of action against the commensal microorganism *Salmonella enterica* subsp. *enterica* ATCC51741 was investigated. The target membrane permeability was determined by detection of beta-galactosidase release from ONPG (o-nitro-phenyl-L-D-galactoside) substrate and changes in the whole protein profile indicating that the peptide extracts destroy the membrane integrity and may induce breaks in membrane proteins to some extent. The release of aromatic molecules such as DNA/RNA was detected after the interaction of *Salmonella* with the peptide extract. Transmission electronic microscopy (TEM) micrographs depicted at least four simultaneous secondary events after the peptide extract treatment underlying their antimicrobial actions, including morphological alterations of the membrane. Spheroplast and filament formation, vacuolation, and DNA relaxation were identified as the principal events from the Gt2 and Cys5-4 peptide extracts, while Gt28 induced the formation of ghost cells by release of cytoplasmic content, filaments, and separation of cell envelope layers. Gel retarding assays indicate that the Gt2 and Gt28 peptide extracts are clearly binding the *Salmonella* DNA, while Cys5-4 partially interacted with *Salmonella* genomic DNA. These results increased our knowledge about the inhibitory mechanism employed by several peptide extracts from native lactic acid bacteria against *Salmonella*. Further, we shall develop peptide-based formulation and evaluate their biocontrol effect in the food chains.

## 1. Introduction

The contamination of food by microorganisms is an overarching problem of the food industry. Salmonellosis causes great harm to the livestock and poultry industries; thus, its effective prevention and control are of great importance to animal husbandry and public health [1]. Efforts to reduce the bacterial growth and the associated diseases along with the expanding bacterial resistance have stimulated research to search for novel antimicrobial agents and related technologies. Several antimicrobial peptides (AMPs) have been isolated from a wide range of species, including single-celled microbes, as they have small molecular weights with good solubility, strong thermal stability, and a broad spectrum of antibacterial activity and are available from a wide range of source materials [2, 3]. To be effective against Gram-negative bacteria, AMPs must be able to disrupt either or both inner and outer membranes and induce apoptosis [4]. AMPs from lactic acid bacteria are promising candidates for the treatment of infectious diseases and food preservation. They usually have a relatively narrow spectrum of inhibition, and their effectiveness towards Gram-negative bacteria depends on the bacterial producer [5, 6]. These peptides are classified according to their structure and properties as follows: (a) class I, called lantibiotics, are very small (>5 kDa) with nisin and lactocin as representatives; (b) class II, called nonlantibiotics, are small (<10 kDa), heat-stable, nonmodified, cationic, hydrophobic peptides containing a double glycine leader peptide, with pediocin PA1, leucocin A, lactococcin G, and plantaricin A as representative of class IIa and class IIb, respectively; (c) class III, larger in size, >30 kDa, are heat-stable peptides with enterolysin A and helveticin J as representative [7]. Within class II, plantaricin EF (PlnEF), plantaricin W, plantaricin JK, and lactacin F are produced by some *Lactobacillus plantarum* species and display antimicrobial activity against both Gram-positive and Gram-negative bacteria [8–10]. Previous research has indicated that some two-peptide bacteriocins induce cell membrane permeabilization or leakage of cellular materials across the cell membrane [10]. Although it was recognized that two-peptide bacteriocins target cellular membranes, there is limited evidence of this mechanism so far [11, 12]; thus, the elucidation of the molecular mechanism of action remains complex [13].

Recently, we showed that the antimicrobial peptide extracts from Gt2, Cys5-4, and Gt28 strains exhibited a broad spectrum of antibacterial activity against several foodborne pathogens; particularly, the mode of action against the commensal microorganisms *Salmonella enterica* subsp. *enterica* ATCC51741, *Escherichia coli* ATCC25922, and *Shigella sonnei* ATCC25931 was investigated. The strains Gt2, Cys5-4, and Gt28 produce two-peptide bacteriocins, such as plantaricin W of Gt2 and Cys5-4 and lacticin 3147, lactococcin M, and lactococcin A of Gt28. Preliminary data indicated that the peptide extract, defined as a mixture of larger size peptides or protein-like components from each individual strain, exerted a bacteriolytic mode of action [14–16]. The efficacy of each peptide extract against *Salmonella* depends on the dose applied, the developmental stage of the target cells, and the incubation time. The Gt28 peptide extract is more effective in inhibition (all bacterial cells were killed upon 3 hours of incubation) than Cys5-4 and Gt2 (marginal inhibitory effect detected at 6 hours of incubation). As the antimicrobial peptide extracts act at the cell membrane, several morphological and ultrastructural changes might occur as the secondary reactions of a live cell to the damage done to its membrane; however, this study was designed to understand the direct injury induced by each peptide extract when individually applied to *Salmonella enterica* on the bacterial envelope or the effect on the intracellular DNA that might explain the microbicidal activity, being a promising approach for biological control of microorganisms in food industry.

## 2. Materials and Methods

### 2.1. Preparation of Partially Purified Peptide Extract and Determination of Minimum Inhibitory Concentration (MIC)

Peptide extracts of Gt2, Cys5-4, and Gt28 from the producer cells of *Lactobacillus plantarum* UTNGt2 (GenBank accession no. KY041688.1), *Lactobacillus plantarum* UTNCys5-4 (GenBank no. KY041686.1), and *Lactococcus lactis* subsp. *lactis* UTNGt28 (GenBank accession no. MG675576.1), respectively, were obtained as previously described [14–16]. Briefly, the overnight bacterial culture was used to extract cell-free supernatant (CFS) by centrifugation at 13,000 × *g* for 30 min (4°C). The CFS was filtered using a 0.22 *μ*m porosity syringe filter (no. STF020025H, ChemLab Group, USA). Then, 60-80% ammonium sulfate was added to precipitate the peptides that were recovered in 25 mM ammonium acetate (pH 6.5), desalted by using a Midi Dialysis Kit (cat no. PURD10005-1KT, Sigma-Aldrich Co. LLC, Saint Louis, MO, USA), preequilibrated with phosphate buffer (pH 7.0), and stored at -20°C before use. Titer, estimated as AU/ml, is defined as the highest dilution that inhibited the growth of the indicator strain. Antimicrobial activity of each peptide was performed using the agar-well diffusion method [17]. The minimum concentration that inhibits 50% of the target was determined as previously described [14]. The MIC was determined as 6400 AU/ml for all peptide extracts.

### 2.2. The Effect of Peptide Extract on Target Cytoplasmic Membrane Permeabilization

*Salmonella enterica* subsp. *enterica* ATCC51741 was maintained as frozen stock cultures in nutrient broth (Difco, Detroit, MI, USA) containing 20% (*v*/*v*) glycerol. The peptide extracts of each strain at concentration 1 X MIC was added to the indicator strain culture (100 ml) at the midexponential phase followed by incubation at 37°C for 24 h. Similarly, the bacterial cells were treated with 0.1% Triton X-100 and used as positive control. To investigate the effect of each peptide extract on membrane permeabilization, the ONPG (o-nitro-phenyl-L-D-galactoside, no. N1127, Sigma-Aldrich Co. LLC, Saint Louis, MO, USA) substrate was used as previously described [16]. The hydrolysis of ONPG to o-nitrophenol (ONP) was monitored at 415 nm at 120 min of incubation. To distinguish between the cytoplasmic enzyme release and peptide uptake to the cells, *β*-galactosidase release was measured from the supernatant [16].

### 2.3. Cell Membrane Integrity Assay

The overnight bacterial suspension of *Salmonella enterica* subsp. *enterica* ATCC51741 washed twice with 1X PBS (phosphate-buffered saline, pH 7.5) were treated individually with 1 X MIC of each peptide extract and incubated for 24 hours at 37°C as described [16]. The cell culture without any treatment was used as control. The DNA/RNA molecules were detected by electrophoresis in 1% agarose gel with ethidium bromide, running in 1X TBE (Tris-borate EDTA, pH 8.0) buffer (Sigma-Aldrich Co. LLC, Saint Louis, MO, USA) after extraction with chloroform (1 : 1, *v*/*v*), and precipitated with isopropanol and ammonium acetate (3 M).

### 2.4. SDS-PAGE of the Target Whole-Cell Proteins after the Treatment with the Peptide Extract

The effect of each individual peptide extract was analyzed using the SDS-PAGE method as previously described [16]. Samples containing *Salmonella* in nutrient broth were incubated independently with 1 X MIC of Gt2, Cys5-4, and Gt28 peptide extract at 37°C for 24 h. The cell pellet was suspended in 1X SDS-PAGE loading buffer, boiled for 5 min at 100°C, and centrifuged at 300 rpm. The supernatants of treated and untreated cells with each peptide extract were used in SDS-PAGE electrophoresis. The tricine-SDS-PAGE method using RunBlue Bis-Tris protein gels (12%) and Dual Cool Mini Vertical PAGE/Blotting Systems (Expedeon, Abcam, Cambridge, MA, USA) was used. The gel was stained with InstantBlue ready-to-use stain (Expedeon, Abcam, Cambridge, MA, USA) using a protocol recommended by the manufacturer.

### 2.5. The Effects of Peptide Extract on the Bacterial Cells under TEM

The test bacteria were treated independently with the peptide extracts at 1 X and 2 X MIC and incubated for 24 h at 37°C. The peptide extract was washed away thrice by using sodium phosphate buffer by centrifuging at 10,000 × *g* for 15 min. The cells were fixed with 2.5% glutaraldehyde and stored overnight at 4°C. The buffers and dehydration protocol used were developed by the Laboratory of Electronic Microscopy, University of Antioquia (Medellin, Colombia). Briefly, the samples were washed thrice with cacodylate buffer and postfixed for 1 h with osmium tetroxide 1% and cacodylate buffer in 1 : 1 ratio. Then, they were washed thrice in cacodylate buffer (10 min) and incubated overnight in the same buffer. The samples were then washed thrice with water, once with uranyl acetate (Sigma-Aldrich Co. LLC, Saint Louis, MO, USA), and again thrice with water. The samples were dehydrated in a graded ethanol series and embedded in Epon (resin). Ultrathin sections were prepared and coated on copper grids and stained with uranyl acetate (Sigma-Aldrich Co. LLC, Saint Louis, MO, USA) and lead citrate (Sigma-Aldrich Co. LLC, Saint Louis, MO, USA). The grids (10 random sections per treatment) were examined using the Tecnai G2 F20 transmission electron microscope (FEI Company, USA). Untreated cells of *Salmonella* were used as control.

### 2.6. Gel Retardation Assay

Genomic DNA of *Salmonella enterica* subsp. *enterica* ATCC51741 was isolated using PureLink Genomic DNA Mini Kit (Invitrogen USA) according to the manufacturer's instruction. The DNA was dissolved in TE buffer (10 mM Tris-HCl, 1 mM EDTA, pH 8.0) at the final concentration 3 *μ*g/*μ*l. The DNA was individually mixed with each Gt2, Cys5-4, and Gt28 peptide extract at the final concentration 1 X MIC in phosphate buffer (pH 7.0) and incubated at room temperature for 60 min. The ratio between the peptide mixture and DNA was 200/3 (*v*/*v*). After incubation, 5 *μ*l DNA mixed with 1 *μ*l 10X loading buffer was analyzed on 1% agarose gel.

## 3. Results and Discussion

### 3.1. The Peptide Extracts of Gt2, Cys5-4, and Gt28 Induced Leakage of Cytoplasmic Content from Salmonella Cells

**Figure 1:**
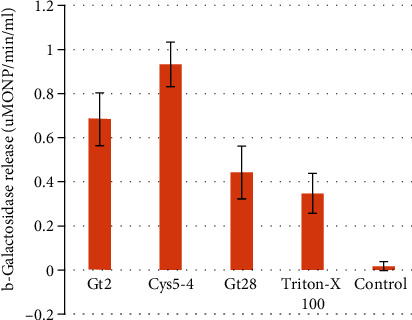
The cytoplasmic membrane permeation of *Salmonella enterica* subsp. *enterica* ATCC51741. Bacteria (after 120 min incubation) were removed by centrifugation, and enzyme release was assayed in the cell-free supernatant. Legend: 1 X MIC of Gt2, 1 X MIC of Cys5-4, 1 X MIC of Gt28 peptide extracts; Triton X-100 (0.1%); control: untreated cells. Results are representative of three independent experiments each made in triplicate. The release of o-nitrophenol (ONP) per minute per milliliter and calculated as described: [A415 × 1000/sample volume (*μ*l)]/reaction time (min) × 4.86, where A415 was the absorbance at 415 nm and 4.86 was the coefficient of extinction (mM^−1^ cm^−1^) of ONP, respectively.

The ability of Gt2, Cys5-4, and Gt28 to permeate *Salmonella* cells was evaluated as a function of cytoplasmic beta-galactosidase release, with bacteria grown in lactose-containing medium. We previously showed that Gt2 has the capacity to increase the *E. coli* cytoplasmic membrane permeability [16]. In this study, the results indicated that the peptide extract of Cys5-4 and Gt2 caused considerable release of the enzyme into the medium at 120 min of incubation (Figure 1). Less membrane permeability was observed with Gt28 and Triton X-100, while no activity was detected in the untreated *Salmonella* cells. Although Triton X-100 is one of the most widely used nonionic surfactants to permeabilize the living cell membrane [18], in this study, it showed lower permeability than Cys5-4 and Gt2 peptide extracts. An early study indicated that a C-type lectin, RegIII*β* (regeneration gene family protein III) protein, enhanced antimicrobial effect towards *Salmonella* typhimurium when the outer membrane integrity was compromised by Triton X-100 [18].

The *Salmonella* cell membrane was compromised after exposure to the peptide extract; thus, a smear DNA with many bands that cannot be easily distinguished along with the RNA molecules was detected in the electrophoresis gel (Figure S1). No DNA/RNA was detected in the untreated sample. Previously, the genomic instability of Gram-negative bacteria induced by antibiotics was observed, but the molecular mechanism was partially understood [19]. In this study, we suggest that the peptide extract possibly might induce double-stranded breaks in the DNA or may interact with target proteins in a manner that might induce partial DNA damage.

### 3.2. Whole Protein Profile of Salmonella enterica ATCC51741 Treated with Peptide Extract

**Figure 2:**
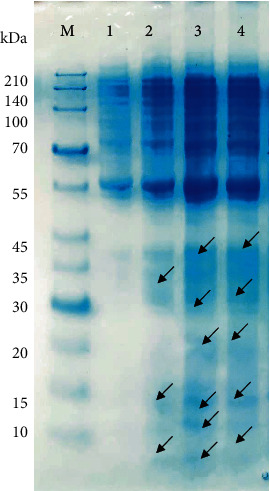
Different expression profiles of *Salmonella enterica* subsp. *enterica* ATCC51741 treated with peptide extract. Legend: lane 1: untreated *Salmonella* (control), lane 2: Gt2, lane 3: Cys5-4, and lane 4: Gt28 at 1 X MIC and 24 h of incubation. Arrows indicate different bands. M: molecular marker (Takara, Clearly Protein Ladder); arrows indicate different bands.

The protein profile depends on the bacterial species, and the interaction with the antimicrobial agents might induce changes or blocking of the protein to expressed [20]. To evaluate the effect of the peptide on the protein profile of *Salmonella*, the cell culture was incubated overnight with the peptides independently. To confirm the membrane permeabilization action of the peptides, *Salmonella* cells were analyzed for soluble proteins by SDS-PAGE. As shown in Figure 2 (lane 1), higher molecular mass proteins were expressed in the lack of peptide extract sample, while in the peptide extract-treated cells, both high- and lower-mass proteins were detected (Figure 2, lanes 2, 3, and 4). The protein profile was distinct, but the identity of the proteins was not further investigated in this study. At this point, we do not know if some bands belong to the peptide itself as they might interfere with the bacterial protein having the same size. An early study indicated that by treating *E. coli* cells with sericin, the bacterial proteins gradually disappeared after 12 h of incubation as shown in SDS-PAGE analysis, which indicated that their expression was blocked [20]. In this study, the peptide extract of Gt2, Cys5-4, and Gt28 may induce breaks of membrane proteins to some extent, this being in concordance with the bactericidal mode of action.

### 3.3. Peptide Extract of Gt2 and Cys5-4 Induced Spheroplast and Filamentous Formation as a Principal Killing Event against Salmonella

**Figure 3:**
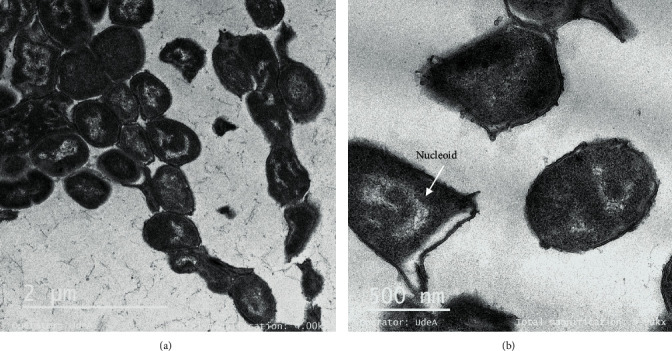
Transmission electron microscopic image of *Salmonella enterica* subsp. *enterica* ATCC51741 cells (exponential phase). Ten different images were taken, and representative images are shown.

**Figure 4:**
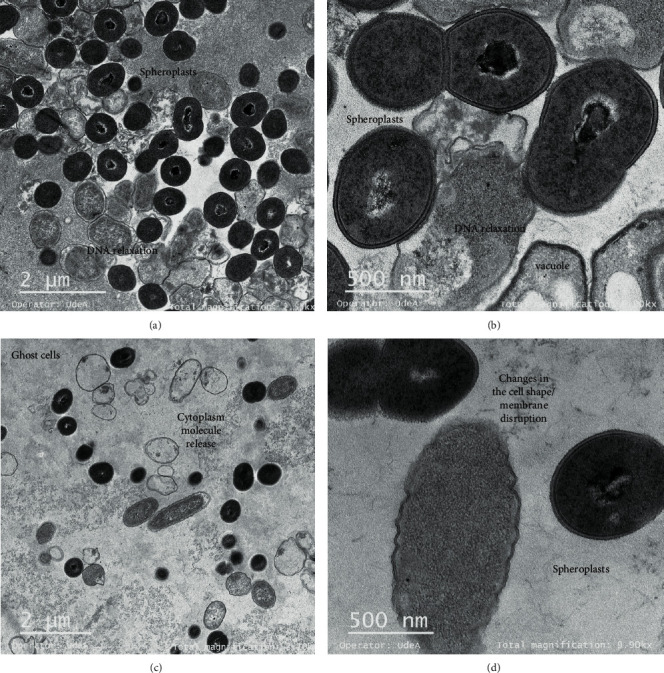
Micrographs of *Salmonella enterica* subsp. *enterica* ATCC51741 treated with Gt2 peptide extract: (a, b) 1 X MIC; (c, d) 2 X MIC. Ten different images were taken for each peptide extract concentration, and representative images are shown.

**Figure 5:**
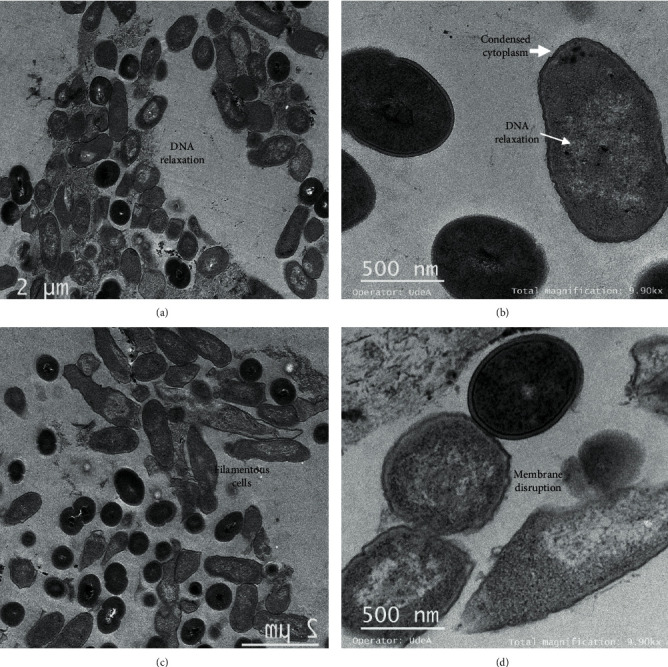
Micrographs of *Salmonella enterica* subsp. *enterica* ATCC51741 treated with Cys5-4 peptide extract: (a, b) 1 X MIC; (c, d) 2 X MIC. Ten different images were taken for each peptide extract concentration, and representative images are shown.

TEM was used to observe the effect of peptide extracts on *Salmonella enterica* after 6 h of incubation. Untreated *Salmonella enterica* cells showed a normal cell shape with an undamaged and intact structure of the inner and outer membrane (Figure 3). By treating *Salmonella* cells with Gt2 peptide extract at the final concentration of 1 X MIC, spheroplast formation was observed (Figures 4(a) and 4(b)). The cells showed changed shape, and the inner and outer membranes were intact, but they lost the peptidoglycan layer. The precise role of peptidoglycans with respect to interaction with antimicrobial peptides is not well understood; they seem to be targets for pathogen recognition [21]. When the concentration of the peptide Gt2 was increased to 2 X MIC, along with the spheroplast, the appearance of some “ghost cells” was noted, indicating that target bacteria were devoid or near-devoid of cytoplasm (Figure 4(c)). The cells presented intact membranes, but the cytoplasm content was released, implying that the Gt2 peptide extract might induce cell death by more than one mechanism.  Table 1 shows a summary of the alteration of the bacterial cell upon the treatment with the peptide extract. Some cells were abnormally longer due to cell elongation or filamentation, the cell membrane changed shape, and intracellular vacuoles were noted (Figure 4(d)). A recent study indicated that cell-free supernatant containing peptides produced by *Lactobacillus taiwanensis* induced ghost cells in *Salmonella gallinarum* [22]. Similarly, vacuole formation increases when the cells are treated with antimicrobials [23]. Treating of *Salmonella* cells with Cys5-4 resulted in spheroplast formation at 1 X MIC (Figures 5(a) and 5(b)), and larger cells with cytoplasmic vacuoles were detected upon the treatment with 1 X MIC (Figures 5(c) and 5(d)). Membrane disruption and DNA relaxation were observed (Figure 5(d)). No ghost cells were detected, indicating that the morphological and ultrastructural changes may rely not only on the concentration and exposure time but also on the identity (origin) of the antimicrobial agent. This mechanism might be explained by the differences in the peptide mixture produced by the Gt2 and Cys5-4 strains; Gt2 has four products of approximately 22, 32, 35, and 55 kDa, while Cys5-4 produces peptides of 10, 15, 20, and 30 kDa [15, 16]. However, more than one peptide or protein-like agent is responsible for the overall antimicrobial activity. Earlier research indicated that antimicrobial agents such as penicillin G, chloramphenicol, oxytetracycline, and kanamycin convert bacteria in spheroplasts [24–26]. In other studies, the beta-lactam induced formation of the spheroplasts in *E. coli* and in many species of Gram-negative bacteria [27]. Most LAB peptides of class II are inhibitory when applied in small concentrations and cause membrane permeabilization and leakage of intracellular components in a sensitive cell [28]. The inhibitory spectrum is limited to Gram-positive bacteria, inducing ion leakage, loss of proton-motive force, and ATP depletion [10]. For example, plantaricin IIA-1A5 produced by *L. plantarum* IIA-1A5 displayed remarkable antimicrobial effects against *S. aureus* by adsorption and attachment onto the cell membrane promoting leakage of the cell membrane with release of organic (proteinaceous and genetic materials) and inorganic (Ca2+, Mg2+, and K+ ions) compounds [29]. Thus far, no spheroplast formation was observed in *Salmonella* cells treated with two-peptide bacteriocins.

### 3.4. Gt28 Peptide Extract Induced Lysis and Ghost Cell Formation

**Figure 6:**
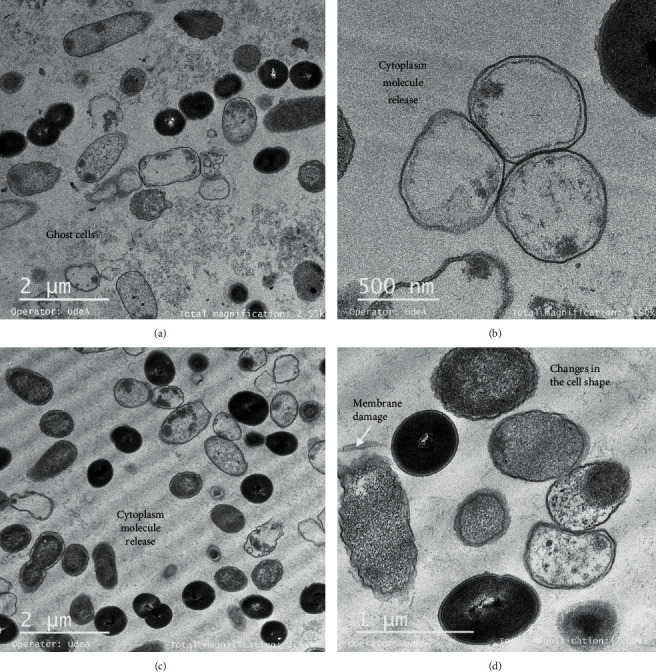
Ghost's cell formation (a–c) and membrane damage (d) after the treatment of *Salmonella enterica* with Gt28 peptide extract at 1 X MIC. Ten different images were taken, and representative images are shown.

**Figure 7:**
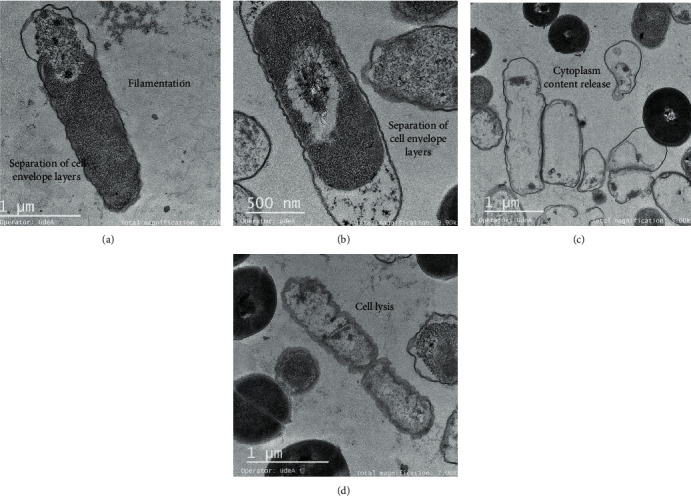
Filamentation and separation of the cell envelope layers (a–c) and ghosts' cells and cell lysis (c, d) after treatment of *Salmonella enterica* with Gt28 peptide extract at 2 X MIC. Ten different images were taken, and representative images are shown.

The UTNGt28 strain peptide extract was more effective against *Salmonella*, and its action was dose-dependent. TEM micrographs of *Salmonella* treated with Gt28 at 1 X MIC caused filamentation, separation of cell envelope layers, and ghost cell formation (Figure 6). Early research indicated that the treatment of *Salmonella enterica* ATCC51741 at both the vegetative and exponential phases of growth with the cell-free supernatant of Gt28 resulted in complete inactivation upon 3 h, suggesting its bactericidal mode of action [14]. Contrary to Gt2 and Cys5-4 peptide extracts containing a mixture of low- and larger weight peptides [15, 16], peptide extract from Gt28 contained one peptide of 15 kDa and some extra larger peptides or protein-like products as deducted in SDS-PAGE, but further analysis is required to detect the extract composition (Figure S2). The cell membrane showed interrupted stretches, and electron-dense material accumulated in the periplasmic space (Figure 6). After the treatment with 2 X MIC, the cell membrane was damaged and the cytoplasmic cell content was released, as many ghost cells were detected (Figure 7). The distance between the cytoplasmic membrane and the outer membrane increased, giving the appearance that the layers of the Gram-negative cell envelope had separated (Figures 7(a) and 7(b)). This effect was shown earlier when studying the action mechanism of several antibiotics such as ciprofloxacin, rifampicin, and vancomycin [23, 30]; this might occur due to the outer membrane detaching from the peptidoglycan. The increase of cytoplasmic membrane release caused by membrane disruption led to leakage of cell cytoplasmic content and cell death. The ghost cell formation indicated lysed bacteria devoid or near-devoid of cytoplasm (Figures 7(c) and 7(d)). Early reports indicated this phenomenon when cells were treated with inhibitors of DNA synthesis [31], RNA synthesis [32], or protein synthesis [33]. The results indicated that a lower dosage of Gt28 peptide extract was sufficient to disrupt the membrane and induce filamentation and separation of the cell envelope layers and cytoplasm leakage. Nonetheless, the extent of killing of bacterial cells was enhanced due to the suprasaturation of cell membrane with peptides. Filamentation can occur following inhibition or disruption of peptidoglycan synthesis, DNA synthesis inhibition, or damage by a SOS response process [34]. This result might explain our recent findings showing the effectiveness of the peptide-based formulations containing Gt28 and Cys5-4 peptide extracts in diminishing the cell viability of a pathogenic cocktail consisting of *Salmonella* sp., *Shigella* sp., and *E. coli* cells at the exponential growth phase [35]. In addition, we demonstrated the grate potential of these peptide-based formulations in the control and protection of pathogenic growth in pineapple fresh-cut chunks.

### 3.5. Gel Retardation Assays Reveal That Gt2 and Gt28 but Not Cys5-4 Peptide Extract Interact with Salmonella Genomic DNA

**Figure 8:**
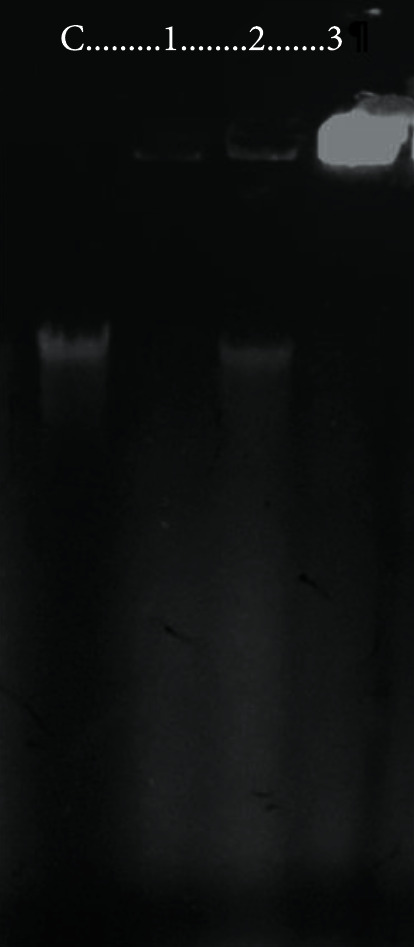
DNA binding analysis of Gt2, Cys5-4, and Gt28 peptide extracts towards *Salmonella enterica* subsp. *enterica* ATCC51741 DNA. C: *Salmonella* genomic DNA; 1-3: genomic DNA treated with Gt2, Cys5-4, and Gt28, peptide extracts.

To identify whether the genomic DNA of *Salmonella* was targeted by the Gt2, Cys5-4, and Gt28 peptide extracts, the genomic DNA was incubated with each peptide extract for 60 min.  Figure 8 shows the agarose gel with the DNA migration. As observed, the Gt2 and Gt28 peptide extracts bind the genomic DNA of *Salmonella*, impeding its migration, while the Cys5-4 peptide extract partially binds the genomic DNA, suggesting that the mode of action of Gt2 and Gt28 but not Cys5-4 might involve binding of negatively charged DNA. Previous investigations indicated that peptides produced by *L. paracasei* subsp. *tolerans* FX-6 do progressively interact with the DNA of *Staphylococcus aureus*, with the complete DNA band retained when the peptides bind DNA [36]. Based on our results, the Gt2 and Gt28 peptide extracts disrupt the target cell, causing damage in the membrane and increasing membrane permeabilization as a secondary effect due to the activation of some autodigestive enzymes; the peptide extracts then enter the cell, might bind negatively charged DNA, and finally cause cell death.

## 4. Conclusions

Taken together, our research showed that the cell membrane of *Salmonella* was permeabilized, peptide extracts possibly induced breaks in membrane proteins to some extent, the cell integrity was lost, and DNA/RNA molecules were released, as well as a direct interaction between DNA and peptide extract occurring, leading to cell death. There were at least four key secondary simultaneous membrane shape changes of *Salmonella* cells induced by the peptide extracts including spheroplast formation, ghost cell formation, cell lysis, and filamentation with separation of cell envelope layers, causing membrane disruption leading to cell death. To our knowledge, this is the first evidence of spheroplasts and ghost cell formation observed as a secondary death event of *Salmonella enterica* subsp. *enterica* ATCC51741 by peptide extracts produced by lactic acid bacteria. Nonetheless, further experimental work will focus on the whole-genome sequencing of these promising antimicrobial strains, allowing to retrieve the gene variants encoding for peptide or protein-like substances responsible for the overall antimicrobial activity, considering the nondairy origin of the producer strains. Finally, these peptide extracts are promising new antimicrobials to enhance the food safety and quality.

## Figures and Tables

**Table 1:** Summary of the various changes that are observed in antibacterial-treated *Salmonella* cells with Gt2, Cys5-4, and Gt28 peptide extracts.

Peptide/concentration applied (MIC)	Alteration	Brief description
Gt2/1 X MIC	Spheroplasts	Spherical bacteria; changed shape, the inner and outer membranes were intact, but they lost of peptidoglycans.
Gt2/2 X MIC	Spheroplasts	Spherical bacteria; changed shape, the inner and outer membranes were intact, but they lost of peptidoglycans.
	“Ghost's cells”	The bacterial cells devoid or near-devoid of cytoplasm.
	Filamentation	Bacteria become much larger than normal.
	Intracellular vacuolation	Round-shaped transparent areas present in the bacterial cytoplasm.
Cys5-4/1 X MIC	Spheroplasts	Spherical bacteria; changed shape, the inner and outer membranes were intact, but they lost of peptidoglycans.
Cys5-4/2 X MIC	Filamentation	Bacteria become much larger than normal.
	Intracellular vacuolation	Round-shaped transparent areas present in the bacterial cytoplasm.
Gt28/1 X MIC	Filamentation	Bacteria become much larger than normal.
	Cell envelope layer separation	An increase in the distance between the cytoplasmic membrane and outer membrane of the gram-negative cell envelope.
	“Ghost's cells”	The bacterial cells devoid or near-devoid of cytoplasm.
	Cell lysis	The cell membrane was damaged leading to leakage.
Gt28/2 X MIC	“Ghost's cells”	The bacterial cells devoid or near-devoid of cytoplasm.
	Cell envelope layer separation	An increase in the distance between the cytoplasmic membrane and outer membrane of the gram-negative cell envelope.

## Data Availability

The data used to support the findings of this study are included within the article.

## Supplementary Materials

Supplementary Materials Figure S1: detection of DNA/RNA molecules released when *Salmonella* was treated with the G2, Cys5-4, and Gt28 peptide extract. Figure S2: the molecular weight of Gt28 precipitated peptide deducted from SDS-PAGE analysis.
