# Supplementary material for: Peptide Extracts from Native Lactic Acid Bacteria Generate Ghost Cells and Spheroplasts upon Interaction with Salmonella enterica, as Promising Food Antimicrobials
Source: Biomed Res Int. 2020 Oct 5;2020:6152356. doi: 10.1155/2020/6152356 (PMC7559518; doi:10.1155/2020/6152356)
Supplement: Supplementary Materials — Figure S1: detection of DNA/RNA molecules released when Salmonella was treated with the G2, Cys5-4, and Gt28 peptide extract. Figure S2: the molecular weight of Gt28 precipitated peptide deducted from SDS-PAGE analysis. [file 6152356.f1.docx]

**Supplementary files**


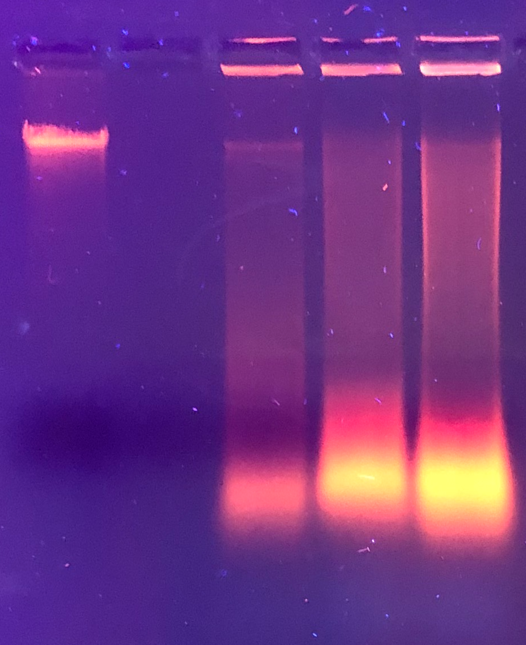


**C. (-). 1. 2. 3**

**Figure S1.** Detection of DNA/RNA molecules released when *Salmonella* was treated with the G2, Cys5-4 and Gt28 peptide extracts. Legend: gDNA- genomic DNA from *Salmonella enterica* subsp. *enterica* ATCC 51741. (-) negative control (no DNA/RNA molecules); 1-3: 1 x MIC of each Gt2, Cys5-4 and Gt28 peptide extract was applied.

**Figure S2**. The molecular weight of Gt28 precipitated peptide deducted from SDS-PAGE analysis.

The tricine-SDS-PAGE method using RunBlue Bis-Tris protein gels (20%) and Dual Cool Mini vertical PAGE/blotting Systems (Expedeon, Abcam, Cambridge, MA, USA) was used. The gel was stained with InstantBlue ready-to-use stain (Expedeon, Abcam, Cambridge, MA, USA) for 4 hours and distained with a solution of 30% methanol (v/v) and glacial acetic acid, 10% (v/v) until the bands became clear. M: molecular marker (Takara, Clearly Protein Ladder); Gt28: purified peptide extract from *L. lactis* UTNGt28 strain
